# Platelets and the Atherosclerotic Process: An Overview of New Markers of Platelet Activation and Reactivity, and Their Implications in Primary and Secondary Prevention

**DOI:** 10.3390/jcm12186074

**Published:** 2023-09-20

**Authors:** Matteo Nardin, Monica Verdoia, Davide Cao, Simone Nardin, Elvin Kedhi, Gennaro Galasso, Arnoud W. J. van ‘t Hof, Gianluigi Condorelli, Giuseppe De Luca

**Affiliations:** 1 Department of Biomedical Sciences, Humanitas University, Via Rita Levi Montalcini 4, Pieve Emanuele, 20090 Milan, Italy; 2 Third Medicine Division, Department of Medicine, ASST Spedali Civili di Brescia, 25123 Brescia, Italy; 3 Division of Cardiology, Ospedale degli Infermi, ASL Biella, 13875 Biella, Italy; 4 Department of Translational Medicine, Eastern Piedmont University, 28100 Novara, Italy; 5 Department of Cardiology, Humanitas Gavazzeni Hospital, 24125 Bergamo, Italy; 6 U.O. Clinica di Oncologia Medica, IRCCS Ospedale Policlinico San Martino, 16132 Genova, Italy; 7 Department of Internal Medicine and Medical Sciences, School of Medicine, University of Genova, 16126 Genova, Italy; 8 Division of Cardiology, Hopital Erasmus, Universitè Libre de Bruxelles, 1050 Bruxelles, Belgium; 9 Division of Cardiology, Ospedale Ruggi D’Aragona, Università di Salerno, 84084 Salerno, Italy; 10 Department of Cardiology, Maastricht University Medical Center, 6229 HX Maastricht, The Netherlands; 11 Cardiovascular Research Institute Maastricht (CARIM), 6229 ER Maastricht, The Netherlands; 12 Department of Cardiology, Zuyderland Medical Center, 6419 PC Heerlen, The Netherlands; 13 Department of Cardiovascular Medicine, IRCCS-Humanitas Research Hospital, 20089 Rozzano, Italy; 14 Division of Cardiology, AOU “Policlinico G. Martino”, Department of Clinical and Experimental Medicine, University of Messina, 98122 Messina, Italy; 15 Division of Cardiology, IRCCS Hospital Galeazzi-Sant’Ambrogio, 20157 Milan, Italy

**Keywords:** platelets, atherosclerosis, platelet reactivity, platelet function test, immunity, genetics, prevention

## Abstract

The key role played by platelets in the atherosclerosis physiopathology, especially in the acute setting, is ascertained: they are the main actors during thrombus formation and, thus, one of the major investigated elements related to atherothrombotic process involving coronary arteries. Platelets have been studied from different points of view, according with the technology advances and the improvement in the hemostasis knowledge achieved in the last years. Morphology and reactivity constitute the first aspects investigated related to platelets with a significant body of evidence published linking a number of their values and markers to coronary artery disease and cardiovascular events. Recently, the impact of genetics on platelet activation has been explored with promising findings as additional instrument for patient risk stratification; however, this deserves further confirmations. Moreover, the interplay between immune system and platelets has been partially elucidated in the last years, providing intriguing elements that will be basic components for future research to better understand platelet regulation and improve cardiovascular outcome of patients.

## 1. Introduction

Platelets enter the circulation after the separation of bone marrow megakaryocytes, as anucleate cells (2 μm in diameter) that lack genomic DNA [1]. Therefore, due to the absence of a nucleus and in the presence of little capacity for protein synthesis, which is only available by messenger RNA (mRNA) transferred from the megakaryocyte, the lifespan of platelets is limited to 7–10 days [2,3]; however, platelets contain mitochondria that have also been transferred from megakaryocyte origin, and therefore, like nucleated cells, they can still benefit from flexible aerobic and anaerobic metabolism in the presence of necrotic and apoptotic pathways that regulate their function and fate [4]. Thrombopoietin is a glycoprotein synthesized mostly in the liver but also in the kidneys, which regulates megakaryocytic proliferation and maturation, as well as platelet production [5]. Once they enter the circulation, platelets have a life span of 7 to 10 days in quiescent discoid state in the presence of healthy vessels without endothelium-impaired homeostasis. Platelets’ primary function is to stop hemorrhage after tissue trauma and vascular damage.

Injury to the intimal layer of the vessel exposes the underlying subendothelial matrix. Platelets move to sites of vascular disruption and adhere to the exposed matrix protein [6]. Adherent platelets undergo activation and subsequently release substances that recruit additional platelets to the site of injury. Additionally, they promote thrombin generation and subsequent fibrin formation. The potent platelet agonist thrombin, together with other amplifying factors, promotes platelet recruitment and activation. Activated platelets then aggregate to form a plug that seals the injury in the vasculature. The understanding of this highly integrated processes is essential to optimize the treatment in patients suffering from atherothrombotic disease [7,8,9]; therefore, the identification of makers of platelet regulation, whether inhibiting or promoting, has deserved increasing attention in the last decades.

## 2. Platelet Adhesion, Activation, and Aggregation

The first step of platelets at sites of vascular injury consists in adhesion, which is mediated by the glycoprotein complexes that allow platelets adhering to exposed factors and collagen. In particular, the glycoprotein (GP) Ib/V/IX mediates the initial capturing of free-flowing platelets binding the immobilized von Willebrand factor (vWF). The induced signals promote platelet main integrin activation, αIIbβ3, which binds fibrinogen at their ligation, which in turn induces a cascade of potent outside signals that significantly increase the cytosolic calcium influx [3]. Mouse models knock-out for the GP VI gene have shown platelets unable to form aggregate and occlusive arterial thrombi [10], and the progressive shedding of GP VI can affect the functional platelets properties of adhesion to the site of injury [11].

The vWF is synthesized by endothelial cells and megakaryocytes assembles into multimers with a size ranging from 550 kDa to more than 10,000 kDa [12]; the prevention of large multimer accumulation is mediated by the metalloprotease ADAMTS13, whose deficiency results in the thrombotic thrombocytopenia purpura.

When released from the α-granules of platelets or the Weibel–Palade bodies of endothelial cells, the majority of the vWF compounds enters the circulation, except for the vWF released from the abluminal surface of the endothelial cells: it accumulates in the subendothelial matrix and binds collagen via its A3 domain. The vWF anchored to the vessel subendothelial matrix changes its conformation, exposing the A1 domain that binds platelets.

The adhesion to collagen and vWF endorses the pathways that lead to platelet activation. The induced signals promote the integrin activation—firstly α2β1, which binds vWF, and then αIIbβ3, which binds fibrinogen. The integrin ligation produces, in turn, a cascade of signals increasing the cytosolic calcium influx [3]. During this process, platelets modify their morphology and stimulate the release of their granule-content-rich soluble agonists, including adenosine diphosphate (ADP), adhesion molecules, and coagulation factors, almost all of which amplify the thrombotic response [13]. Among these autocrine and paracrine mediators, platelet activation favors thromboxane A2 (TXA2) generation through cyclooxygenase1. The next step involves the conversion of prothrombin into thrombin through activated factor X [14]. Thrombin is the most potent known agonist for platelet activation, binding protease-activated receptor types 1 and 4 (PAR-1 and PAR-4), but it also plays a critical role in early thrombus formation, converting fibrinogen to fibrin, which effectively anchors the growing thrombus.

**Figure 1:**
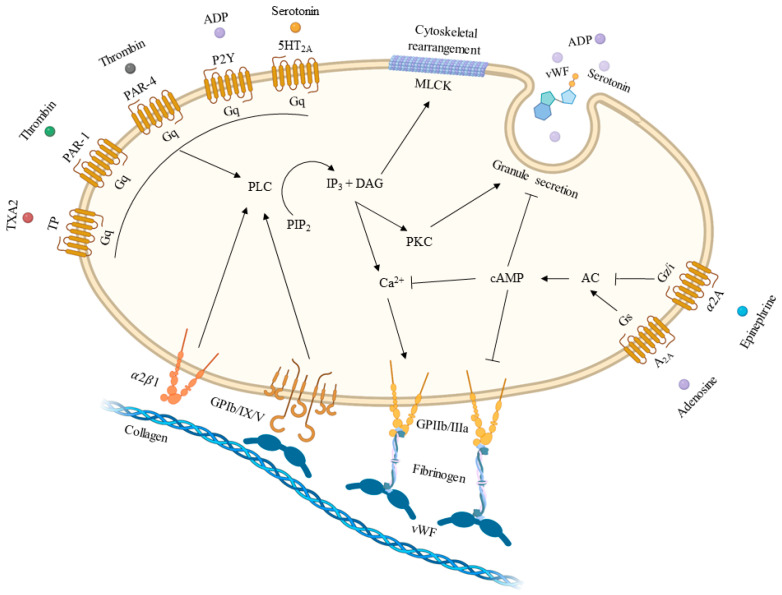
**Platelet activation pathways.** The figure displays the intricate relationship between different stimuli and platelet activation. Created with BioRender.com. AC = adenylate cyclase; ADP = adenosine diphosphate; cAMP = cyclic adenosine monophosphate; DAG = diacylglycerol; IP3 = inositol triphosphate; MLKC = myosin light chain kinase; PAR-1 = protease activated receptor-1; PAR-4 = protease activated receptor-4; PIP2 = phosphatidylinositol bisphosphate; PKC = protein kinase C; PLC = phospholipase C; TP = thromboxane receptor; TXA2 = thromboxane A2; vWF = von Willebrand factor.

The final step in the formation of the platelet plug is represented by the aggregation that links platelets to each other to form clumps. Linkages are mediated by GP IIb/IIIa that has undergone conformational change after platelet activation to increase the affinity for its ligand, fibrinogen (Figure 1) [15].

## 3. Interplay Immune System

**Figure 2:**
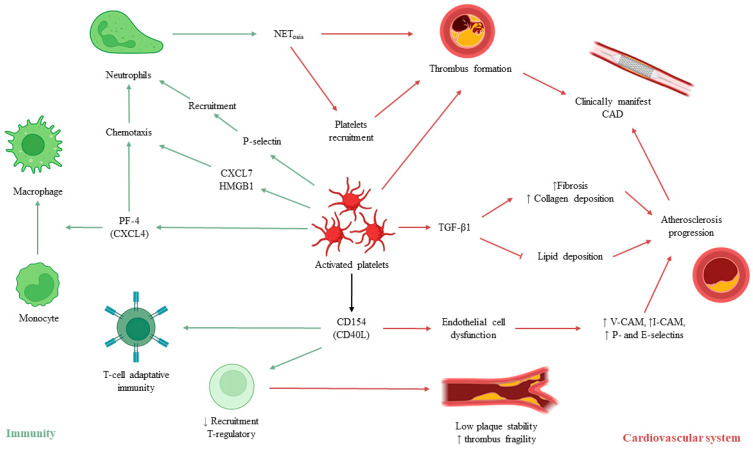
**Activated platelet interplay between immune and cardiovascular systems.** The figure shows complex interplays involving activated platelets between immune and cardiovascular system: on the left side, crosstalk between platelet and immune system is shown, reporting principal signaling pathways. On the right side, the main cardiovascular actions played by activated platelets are reported. The lines with arrows indicate promotion/activation, while the lines with final bar indicate blockage/inhibition. Created with BioRender.com. CAD = coronary artery disease; CXCL7 = chemokine (C-X-C motif) ligand 7; HMGB1 = high mobility group box 1; I-CAM = Intercellular adhesion molecule; NET = neutrophil extracellular trap; PF-4 = platelet factor-4; TGF-β1 = transforming growth factor-β1; V-CAM = Vascular adhesion molecule.

The immune system has a central key role in the atherothrombotic process [16,17]. Platelets have been involved in modulation of innate and adaptive immune responses, even if most mechanisms are still an enigma (Figure 2). Platelets α-granules contain many molecules with several effects not limited to platelet aggregation amplification and coagulation: a couple of molecules with a key role in several signaling pathways are CD40 and CD154, also known as CD40 ligand (CD40L) [18]. In particular, CD154 can interact both with endothelial and immune cells. The stimulation of endothelial cells leads to increased expression of adhesion molecules, mainly intercellular (I-CAM-1) and vascular (V-CAM-1) adhesion molecules [19], and consequent increased leukocytes recruitment and atherosclerotic plaque instability. Platelet-derived CD154 interacts with different type of immune cells: it has been described stimulating the B cell differentiation [20] and macrophages activity [21]. In a murine model of atherosclerosis, CD154 has been shown to increase plaque fragility and reduce its stability by inhibition of regulatory T cells [22]. Platelets have been found expressing different toll-like receptor (TLR) subtypes, enforcing the concept of inflammation modulatory action by platelets [23]. Among cytokines released by platelets, transforming growth factor-β1 (TGF-β1) is one of the most important and studied. It has been characterized with both anti-atherogenic and pro-atherogenic effects, given its number of potential targets. Briefly TGF-β1 may prevent the formation and progression of endothelial lipidic deposit by increasing the nitric oxide synthetase activity and polarization of macrophage into anti-inflammatory phenotypes [24], with consequent enhanced plaque stability [25]. The TGF-β1 pro-atherogenic effect has been elucidated in studies reporting its promotion of interstitial collagen deposition, fibrosis, and blockade of endothelial regeneration, with consequent growth of lesion and reduction in vascular lumen [26].

Moreover, platelets have been described to have the opportunity to recruit neutrophils and worse thrombo-inflammation [27].

In fact, leukocytes’ interaction with the endothelium is mediated by the different types of surface-expressed selectins and their specific ligands. Among selectins, E-selectin expression mainly occurs in presence of endothelial dysfunction [28] while P-selectin is expressed on both endothelial cells and platelets upon activation [29]. A damaged endothelium is rapidly covered by activated platelets which in turn capture circulating neutrophils into thrombus: in murine models, knock-out for P-selectin showed marked lower or absent leukocyte recruitment [30]. The main crosstalk element is constituted by the P-selectin that binds P-selectin glycoprotein ligand-1 expressed by neutrophils [31], but also chemokines CXCL4 (also named platelet factor-4, PF-4) and CXCL7 (also named neutrophil activating peptide, NAP-2) [32], and high mobility group box 1 (HMGB1) are released by platelets and contribute to neutrophil chemotaxis [33]. Neutrophil β2 integrin binds PF-4, while NAP-2 attracts and stimulates neutrophils through the engagement of CXCR1 and CXCR2 [34].

A further step of neutrophil–platelet interactions is the promotion of neutrophil extracellular trap (NET) [35,36]. This structure consists of extracellular chromatin supported by the histones. Activated platelets have been demonstrated inducing a rapid release of NET, not only using the P-selectin-dependent interplay, but also the TLR4 pathways, like in septic condition [37]. Moreover, NET itself may induce the thrombus formation, serving as a scaffold for both platelet binding and activation [38]. Several pathways have been suggested, including H3 histones and C3b attached to NET [39]: in murine models, the infusion of histone preparate was related to accelerated thrombus formation and vWF release [40]. Formed NET can reduce the endothelial integrity, which in turn determines platelet recruitment and activation and coagulation through factor XII, as well as NET physical interaction with activated fibrinogen providing stability to the thrombus [41,42].

From the clinical side, Hally et al. have investigated the potential utility of a composite biomarker score of NET activation and release, or NETosis, for predicting major cardiovascular adverse event (MACE) post-myocardial infarction (MI): authors demonstrated the importance of these combining biomarkers as risk predictors of MACE at 1 year [43]. Furthermore, Riegger et al. evaluated about two hundred and fifty thrombus specimens in patients with stent thrombosis, demonstrating the recruitment leukocytes, particularly neutrophils and the presence of NETosis in 23% of samples [44]. In the thrombus of culprit artery from patients with acute MI, the presence of NET formation has also been demonstrated [45]. Promising therapeutics have been investigated, inclacumab and crizanlizumab, that block interaction of P-selectin and neutrophils. In the SELECT-ACS study, inclacumab was reported to significantly reduce myocardial damage assessed through the CK-MB after percutaneous coronary intervention (PCI) in patients with non-ST segment elevation acute coronary syndrome (ACS) [46,47]. However, these findings were not confirmed in patients suffering of bypass graft failure after coronary artery bypass surgery [48]. Therefore, further studies are needed to better assess the potential of monoclonal antibodies therapy against NET in patients with coronary artery disease (CAD) [49].

## 4. Morphologic and Structural

Among the first proposed parameters linked to platelet aggregation, there is the mean platelet volume (MPV). MPV is a marker of platelet size, and it is usually provided by the majority of laboratories as a part of the full blood count [14]. It constitutes one of the most used indirect markers of platelet function. In fact, it has been shown that larger platelets are more active, from a metabolic point of view, thus leading to greater prothrombotic risk [50,51]. Early released platelets display higher MPV, while aging decreases platelet volume due to microvesiculation of fully activated platelets or apoptotic membrane fragmentation [52].

In their denser granules, larger platelets contain more β-thromboglobulin, serotonin, and TXA2 than smaller ones [53,54,55]. Moreover, it has been argued that MPV may reflect the platelet production rate [56], stimulation [57], and activation [58,59,60]. In particular, larger platelets are more adhesive and reactive [61,62], showing higher expression of glycoprotein Ib and GP IIb/IIIa receptors at the surface [63]. For these reasons, MPV has been variously associated to CAD, although with contrasting results [64]. In about 400 patients with ST segment elevation MI undergoing primary PCI, Huczek et al. found MPV to be a strong, independent predictor of impaired angiographic reperfusion and six-month mortality [65]. Similarly, Murat and colleagues showed MPV as an independent predictor of the severity of CAD among ACS patients [66].

However, the direct relationship between MPV and CAD has not been validated by other investigations [67,68]. Halbmayer et al., in fact, found no differences in MPV between healthy persons and patients with CAD as well as no significant variations of MPV values being reported between patients without prior MI and MI survivors [69]. In a large cohort of more than 1400 patients undergoing PCI, no relationship was found between MPV and the extent of CAD [70], neither with platelet aggregation [71].

Whether MPV might represent an independent predictor of cardiovascular (CV) events or on the contrary the consequence of other CV risk factors, such as diabetes, hypertension, or smoking is still debated, even if a recent meta-analysis suggests that MPV could be a useful prognostic marker in patients with CAD [72].

Another morphologic parameter of platelets that has been inquired upon for a potential relationship with CAD is platelet distribution width (PDW): it directly measures the variability in platelet size and has been used to differentiate primary disorders of platelets such as essential thrombocythemia from reactive thrombocytosis [73], therefore providing more information than MPV. Some studies have described a potential relationship between PDW and chronic total occlusion of CAD [74] and occluded saphenous vein graft in patients with coronary artery bypass graft surgery [75]. Opposite results were reported regarding the role of PDW in predicting CAD and its severity, suggesting the need of further investigations to clarify its contribution in patient management [76,77].

In addition, the platelet-large cell ratio (P-LCR), an index representing the percentage of platelets larger than 12 fL, has deserved attention as a marker of platelet activation [78]. Despite some promising results showing a relationship between P-LCR and inflammation in CAD patients [79,80], no significant contribution regarding the severity of CAD and platelet reactivity has been found for P-LCR [81,82].

Growing interest has been denoted about the fraction of reticulated platelets, which represent the proportion of younger platelets released from the bone marrow last, with a higher content in α-granules and RNA: these features have been hypothesized as leading to enhanced capability of proteins synthesis and then to a potentially increased overall reactivity [83,84]. McBane et al. reported that younger reticulated platelets appear to have a greater propensity for thrombus participation in presence of atherosclerotic stenosis and shear conditions compared to older ones. Among the suggested mechanisms, an increased receptor density of integrin-β3 in younger platelets may contribute to higher predisposition to thrombosis [85].

Immature platelet fraction (IPF) and the absolute immature platelet count (IPC) represent parameters for the identification of the reticulated platelet with a sample for a routine blood count [86]. A certain number of studies has reported higher IPF in patients with CAD, especially in the subgroups admitted for an ACS [87,88], with a potential role of IPF > 6.2 to predict mortality [87]. On the other hand, Berny-Lan et al. found no difference of IPF in patients with and without ACS [89] and Verdoia et al. showed no role of IPF to predict CAD and the severity of CAD in patients undergoing PCI, discussing IPF as a marker of platelet turnover rather than being directly involved in the pathogenesis of CAD. In patients on antiplatelet drugs, IPF has been linked with ineffective therapy, both mono- and dual-antiplatelet therapy [90,91], despite opposite evidences having also been provided [92,93].

## 5. Function and Reactivity

A direct assessment of platelet activation and aggregation could be performed though a platelet function test (PFT), of which the most used are displayed in Table 1. The identification of impairment in clot formation for hemorrhagic disorders was the rationale of the first test described by Duke, the evaluation of bleeding time [94]. To better assess congenital and acquired platelet disorders, the first platelet aggregation test was developed, adopting the light transmission aggregometry (LTA) with platelet-rich plasma (PRP) [95,96]. After them, several tests with different methods have been proposed to estimate platelet function. Apart from LTA, which requires elaborate management of blood samples before proceeding, impedance whole aggregometry allows to assess platelet aggregation using anticoagulated whole blood. It allows to measure platelet aggregation after several stimuli inquiring different activation signaling pathways with two main advantages: the small quantity of whole blood required and no manipulation before testing, preserving the physiological condition as much as possible [97]. Another aggregometry method uses a turbidimetric-based optical detection through a system cartridge containing fibrinogen-coated beads and platelet agonists [98]; specific assays for patients under antiplatelet therapy are commercially available [99]. A certain number of commercial assays are instead based on the evaluation of platelet adhesion under shear stress, or on viscoelastic methodologies [100,101,102,103].

Besides the different technologies used, the rationale of assessment of platelet function in patients with CAD is used for monitoring the response to antiplatelet drugs in order to identify subjects deserving modification in the composition and/or treatment duration [104]. The main reason of assessment is to recognize patients that are poor responders to antiplatelet therapy, both aspirin and P2Y_12_ inhibitors. Since these drugs represents the cornerstone in patients with CAD, undergoing PCI, their effectiveness is crucial to prevent adverse events [105]. However, the adoption of the expression “drug resistance” is not appropriate, considering that the baseline assessment of platelet function is unavailable in the vast majority of cases, especially in the acute setting. Therefore, it is recognized to refer to inappropriate platelet reactivity during antiplatelet therapy with the expression of “high residual platelet reactivity” (HRPR) or “high on-treatment platelet reactivity” (HTPR) [106]. Even if HRPR is a well-known independent predictor of several adverse events [107], strategies to tailor antiplatelet therapy according to platelet function provided contrasting results in demonstrating clinical benefits [104,108,109,110], suggesting other factors involved in the determinism of elevated platelet reactivity on top of antiplatelet treatment (Table 2).

## 6. Platelet Reactivity

The detection of patients displaying enhanced platelet reactivity despite antiplatelet therapy and deserving more clinical attention and stricter follow-up could improve the overall outcome of those subjects at higher risk for CV adverse events.

One of the first described elements affecting atherosclerosis and platelets is cigarette smoking. The impact on the CV system is recognized, while less detailed are the smoking mechanisms involved in the promotion of platelet reactivity. The increased smoking-induced lipid oxidation products bind platelet scavenger receptor CD36, which in turn results in increased platelet aggregation response [124]. Amplifying factors, including ADP and thrombin, have been shown to increase after cigarette smoking exposure, with consequent higher platelet aggregation response to these pathways [125,126], and nicotine displayed a direct role in increasing platelet aggregability [127]. However, some recent studies have found an opposite effect of smoking, especially on P2Y_12_ antagonists, whose platelet inhibition is enhanced after smoking exposure [128], despite concerns regarding basal lower platelet aggregation [129] and smoking effect on platelet morphology [130].

Higher platelet reactivity has been reported among diabetic patients, due to continuous environmental inflammation that characterizes diabetes mellitus. However, diabetes is not only per se a marker of higher risk [131], but the impaired glycemic control has also been linked to HRPR among clopidogrel-treated subjects [132] or under more potent P2Y_12_ inhibitors [133]. Conversely, Vivas et al. documented a significant reduction in aggregation in post-ACS patients receiving intensive glucose control treatment with insulin [134]. Indeed, hyperglycemia can impact platelet function both directly and by modulating the release of pro-oxidant and inflammatory substances or by the glycation platelet surface proteins, with consequent amplification of platelet adhesion [134].

Similar pathophysiological elements are shared by the excess of uric acid: it has been addressed as a main determinant of atherosclerosis and metabolic syndrome. In fact, hyperuricemia is a condition characterized by impaired nitric oxygen release and enhanced pro-inflammatory cytokines [135,136]. However, no direct impact has been demonstrated on platelet reactivity under antiplatelet therapy [137].

Increasing interest has grown regarding the potential CV impact of vitamin D [138]. Alongside the impact on endothelial dysfunction [139], a vitamin D receptor was detected on platelet surface, displaying antithrombotic effects [140]. Pivotal role in the platelet aggregation and thrombus formation has been described by Aihara et al., who demonstrated in murine models knock-out for the vitamin D receptor gene an enhanced thrombogenicity [141]. An indirect antiplatelet therapy has been previously reported by Lòpez-Farré and colleagues: the addition of vitamin D binding protein to whole blood in healthy subjects hampered the antiplatelet inhibitory effect of aspirin [142], and lower levels of vitamin D have been significantly associated to HRPR under ticagrelor treatment [143]. Genetics of vitamin-D-related genes constitutes an interesting ongoing field of investigation [144], despite more studies being needed [145].

## 7. Genetics and miRNAs

In relation to the antiplatelet therapy with P2Y_12_ inhibitors, several reports have addressed concerns on reduced effectiveness and platelet inhibition, especially during clopidogrel treatment. Because clopidogrel is a pro-drug, it first needs a metabolic modification by CYP2C19 before it becomes effective. However, about 5% of Caucasians and 15 to 20% of Asians display low or absent CYP2C19 activity, leading to a smaller or no clopidogrel effect on platelet function [146]. Several studies have reported higher number of CV adverse events in subjects who carry at least one non-functional copy of the CYP2C19 gene compared with patients with wild-type CYP2C19 gene [147,148], portraying these patients to be treated with higher clopidogrel dosage or with an alternative drug [149,150].

Guidelines to describe scenarios deserving modification in antiplatelet therapy have been proposed by the Clinical Pharmacogenetics Implementation Consortium (CPIC) and by Dutch Pharmacogenetics Working Group (DPWG) of the Royal Dutch Association for the Advancement of Pharmacy [151,152]. However, the recent TAILOR-PCI trial showed that in CYP2C19 loss of function carriers with ACS and stable CAD undergoing PCI, genotype-guided selection of an oral P2Y_12_ inhibitor, compared with conventional clopidogrel therapy without genotyping, resulted in non-statistically significant difference with respect to a composite end point of CV death, MI, stroke, stent thrombosis, and severe recurrent ischemia at 1 year [153], while a meta-analysis suggests the potential usefulness of genetic testing to guide de-escalation of antiplatelet therapy after a PCI for ACS [154].

**Figure 3:**
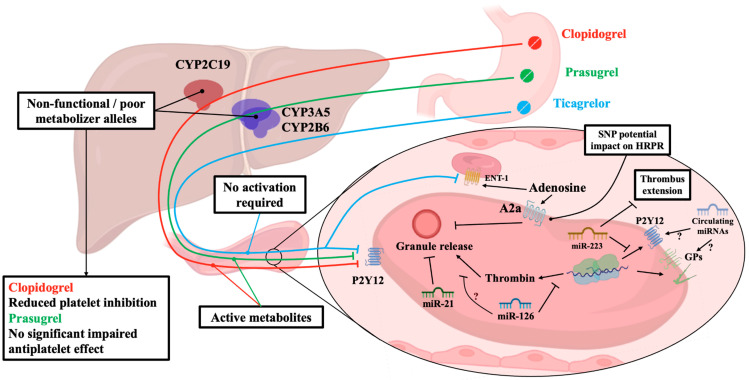
**Genetic and miRNAs.** The figure depicts essential but crucial steps in pharmacokinetic of routinely used P2Y_12_ inhibitors, coupled with the potential target of the most studied miRNAs. The lines with arrows indicate promotion/activation, while the lines with final bar indicate blockage/inhibition. Created with BioRender.com. GPs = glycoproteins; ENT-1 = equilibrative nucleoside transporter-1; HRPR = high residual platelet reactivity; miRNAs = micro-RNAs; SNP = single nucleotide polymorphism; ? = hypothesized action.

In addition, more potent and expensive P2Y_12_ inhibitors were inquired for a potential impact of genetics on their effects: prasugrel, a pro-drug like clopidogrel, was found being influenced by some cytochrome P450 polymorphisms [155], even if the Food and Drug Administration stated that there were no relevant effects of genetic variation on pharmacokinetics of prasugrel’s active metabolite and, thus, on its antiplatelet effect [155]. On the other hand, ticagrelor, that is direct P2Y_12_ inhibitor not requiring activation, has no specific warning about potential genetic impact: Varenhorst et al. found some genetic loci influencing ticagrelor pharmacokinetics through a genome-wide association study, but that did not translate into any clinical detectable effect on ticagrelor efficacy and safety [156], even if the genetics of the adenosine signaling pathways may affect platelet reactivity under ticagrelor treatment (Figure 3) [157].

In the last years, increasing interest has been dedicated to RNA carried by platelets, specifically with regards to microRNAs (miRNAs). Considering that platelets are enriched with miRNAs and represent the second most abundant blood cell type, platelets are the major source of miRNAs in plasma and serum [158,159]. Moreover, the levels of both intraplatelet and circulating platelet-derived miRNAs are shown to correlate with platelet reactivity [160,161].

Of about 750 intraplatelet miRNAs identified, the most extensively investigated are miR-223, miR-21, and miR-126. With respect to the overall amount of platelets, miR-223 is the most represented, and it has been linked to P2Y_12_ receptor regulation, despite the exact mechanism needing to be clarified [162]. However, some suggestions come from murine model without miR-223 expression, which displayed increased thrombus size compared to normal miR-223 expression [163]; among diabetic patients, a lower level of miR-223 was detected with a concomitant increased platelet reactivity [164]. On the other hand, miR-21 together with other miRNAs were found upregulated in ACS patient with an enhanced response to clopidogrel [165]; however, this deserves further study to define its role in regulation of α-granules and platelet-derived pro-fibrotic factors, including TGF-β1 [166]. The role of miR-126 has been shown involving the regulation of P2Y_12_ receptor and, potentially, platelet-derived thrombin generation [167,168].

The role of circulating miRNAs in platelet activation is still under investigation, even if a progressive quantity of evidence has been published, underscoring their role in aggregation homeostasis [169,170]. Major shadows are related to the general mechanisms used by circulating miRNAs to act on their target, especially considering the abundance of RNases in the circulation that quickly degrade free RNA [171]: it has been proposed that circulating miRNAs might regulate the cell surface receptor through physical conformational interaction, but an adversative issue is that miRNAs are contained in vesicles or protein complexes to protect them by degradation [172]. Jansen et al. have described in 181 patients with stable CAD that miRNAs contained in microvesicles but not circulating miRNAs predict the occurrence of CV events [173]. In addition, Zampetaki et al. described the association of miRNAs expression patterns and the incidence of MI in a cohort of 820 patients [174], while other miRNAs levels were significantly associated with the risk of death in univariate and age- and sex-adjusted analyses in patients with ACS [175]. Further investigations will provide us a more detailed understanding of miRNAs pathways to design accurate studies for the definition of their functions in platelet aggregation and atherosclerosis progression.

## 8. Future Perspective and Conclusions

This overview aimed to show the different approaches and methods adopted to study platelet aggregation that appropriately warranted the attention of scientific research: not only because they are an essential component of the physiological hemostasis and pathological atherothrombotic process, but also since interventions on them have demonstrated dramatic improvements in patients with CAD, including both stable disease and acute presentation. Antiplatelet therapy represents a mandatory approach for subjects with clinically significant atherosclerosis, but the availability of effective parameters to assess platelet function is indispensable to select the best option for each patient in the era of a tailored therapeutic approach [176,177,178]. From the first instrument developed and of limited routinary usage, several morphological parameters and methods to assess platelet reactivity have shown significant contribution to daily clinical practice. Auspicious findings are from genetic investigations, in particular miRNAs, as well as from the progressively better understanding of the role of immune system cells in platelet thrombus formation. A single, definite biomarker of platelet aggregation would probably be chimeric, whilst the merging of different aspect assessments, including the most recent advances, will help—if not to close, then at least to move near the circle on a comprehensive valuation of the CV risk for patients with CAD.

## Figures and Tables

**Table 1:** Main platelet function test used in clinical practice.

Methods	Sample	Application	Principle	Advantages	Disadvantages
**Tests based on platelet aggregation**					
Light transmission platelet aggregation (LTA); also named optical platelet aggregation	Citrated PRP	Screening test for bleeding propensity Diagnostic for platelet defects, both congenital and acquired Monitoring antiplatelet treatment effect	Photo-optical measurement of light transmission increase in relation to specific agonist-induced platelet aggregation	Historical gold standard Diagnostic method Different platelet pathways investigated Sensitive to different antiplatelet drugs therapy	Manual and long sample processing Pre-analytic and analytic variables High sample volume Time consuming
Impedance platelet aggregation	Citrated WB	Screening test for bleeding propensity Diagnostic for platelet defects, both congenital and acquired Monitoring antiplatelet treatment effect	Measurement of electrical impedance between two electrodes after induction of platelet aggregation through a specific agonist	No sample processing Diagnostic method Flexible Different platelet pathways investigated Sensitive to anti-platelet therapy Close to LTA	Sample preparation Time consuming
Lumiaggregometry (i.e., VerifyNow)	Citrated WB	Modified aggregometry for detection of storage/release disorders	LTA or WB aggregometry combined with luminescence	No WB processing Quick and easy to do methods Monitoring antiplatelet therapy	Nonflexible Very expensive Limited hematocrit and platelet count
Plateletworks	Citrated WB	Monitoring of the platelet response to antiplatelet agents	Platelets’ counting and aggregates pre- and post-activation (use of ADP or arachidonic acid) in whole blood based in GP IIb/IIIa-dependent aggregation	Minimal sample preparation Easy, rapid screening test	Indirect assay Performance within few minutes after sample collection Required adjunctive platelet count Scarce clinical data
**Platelet function methods combined with viscoelastic test**					
TEG/r-TEG platelet mapping system	Citrated WB	Assessment of global hemostasis plus monitoring antiplatelet treatments effect	Assessment of the rate and the strength of clot formation based on low shear-induced and agonist addition	Point of care for viscoelastic test Global hemostasis test Measure clot properties Reduces blood transfusions	More studies are needed
ROTEM platelet	Citrated WB	Assessment of global hemostasis plus diagnostic of platelet defects plus monitoring antiplatelet treatments effect	Measurement of electrical impedance increase in relation to agonist-induced platelet aggregation	Adaptation from TEG: results are identical Predicts bleeding Reduces blood transfusions Improves clinical outcome Global hemostasis test WB platelet aggregometry	Limited hematocrit and platelet count range (for platelet system) Lack of clinical studies
**Tests based on platelet adhesion under shear stress**					
PFA-100; Innovance PFA-200	Citrated WB	Assessment of bleeding risk and drug effects Searching severe platelet dysfunctions, revealing of vWF disease	Time evaluation of high shear WB flow blocked by platelet plug into a hole in activated surface. Use of combination of collagen/epinephrine or collagen/ADP	In vitro standardized bleeding test Quick and easy to do Sensitive to severe platelet defects	Rigid closed system Dependent on hematocrit, platelet count and vWF Not sensitive to platelet granules defects Contrasting evidence in thienopyridines treatment, especially for PFA-100
IMPACT—Cone and Plate(let) Analyzer (CPA)	Citrated WB	Screening of primary hemostasis and platelet defects	Shear-induced platelet adhesion–aggregation upon specific surface covered by polystyrene. Ongoing studies on addition ADP and arachidonic acid for antiplatelet therapy monitoring	Global platelet method Small sample volume	Expensive Experienced staff Lack of clinical studies Not widely available
**Platelet analysis based on flow cytometry**					
Flow cytometry	Citrated WB, PRP, washed platelets	Cell counting, detection platelet activation by extent of expression of surface and/or cytoplasmic biomarkers	Engineering laser-based detection of suspending fluorescent label platelets in a flowing solution	Useful into diagnose inherited platelet disorders	Expensive Experienced staff Not widely available
Vasodilator Stimulated Phosphoprotein (VASP)	Citrated WB	Intracellular platelet pathway	Immunofluorescence on assay with a specific monoclonal antibody	Useful into monitoring antiplatelet drug	Expensive
**Evaluation of Thromboxane metabolites**					
Radio- or enzyme-linked immune assays	Serum, urine, citrated plasma	Measurement of TXA2 metabolites (and β-TG, PF4, soluble P-selectin)	Ligand-binding assays	Directly related to COX-1, the aspirin’s target	Indirect measure No platelet specific

ADP = adenosine diphosphate; β-TG = β-thromboglobulin; COX-1 = cyclooxygenase-1; LTA = light transmission platelet aggregation; PF4 = platelet factor-4; PFA = platelet function assay; PRP = platelet-rich-plasma; ROTEM = rotational thromboelastometry; r-TEG = rapid thromboelastography; TEG = thromboelastography; TXA2 = thromboxane A2; WB = whole blood; vWF = von Willebrand factor.

**Table 2:** Main clinical trials using platelet function test for tailoring antiplatelet therapy.

**Study**	**Year**	**PFT**	**Intervention**	**Subjects**	**Study design**	**Findings**
VASP-02 [111]	2008	VASP	VASP-guided switch after 2 weeks in low responders	150 patients undergoing elective PCI	Randomized to 150 mg vs. 75 mg clopidogrel	Greater platelet inhibition with 150 mg clopidogrel in poor responders
Bonello et al. [112]	2008	VASP	VASP-based adjustment of clopidogrel loading dose	162 patients undergoing PCI with basal VASP > 50%	Randomized to VASP-guided vs. standard of care	Lower rate of 1-month MACE in VASP-guided group
Cuisset et al. [113]	2008	LTA	PFT-based identification of poor responder before randomization	149 clopidogrel non-responders undergoing elective PCI	Randomized to administration of additional GP IIb/IIIa antagonist vs. standard of care	Lower rate of 1-month CV event in intervention group
3T/2R [114]	2009	VerifyNow	PFT-based identification of poor responder before randomization	263 ASA and/or clopidogrel non-responders undergoing PCI	Randomized to tirofiban administration vs. standard of care	Lower MI and 1-months MACE in intervention group
Wang et al. [115]	2011	VASP	PFT-based drug adjustment in tailored strategy	306 patients undergoing PCI with basal VASP > 50%	Randomized to VASP-guided vs. standard of care	Lower rate of 1-year MACE in VASP-guided group
GRAVITAS [116]	2011	VerifyNow	PFT-based identification of poor responder before randomization	2796 clopidogrel non-responder patients undergoing PCI	Randomized to higher dose of clopidogrel vs. standard of care	No differences in 6-month MACE rate
Aradi et al. [117]	2012	LTA	PFT-based identification of poor responder before randomization	200 clopidogrel non-responders undergoing elective PCI	Randomized to higher dose of clopidogrel vs. standard of care	Lower MI and 1-months MACE in intervention group
Hazarbasanov et al. [118]	2012	Impedance aggregometry	PFT-based drug adjustment in tailored strategy	192 patients undergoing PCI	Randomization to tailored strategy vs. standard of care	Lower rate of 6-months MACCE in intervention group
TRIGGER-PCI [119]	2012	VerifyNow	PFT-based identification of poor responder before randomization	423 clopidogrel non-responders undergoing PCI	Randomized to switch to prasugrel vs. clopidogrel maintenance	Higher platelet inhibition in prasugrel group.
ARTIC [120]	2012	VerifyNow	PFT-based administration of additional bolus of clopidogrel, prasugrel or ASA along with GP IIb/IIIa antagonist	2440 patients undergoing elective PCI	Randomization to tailored strategy vs. standard of care	No differences in 1-year MACE
MADONNA study [104]	2013	Impedance aggregometry	PFT-based additional antiplatelet drug loading dose	798 patients undergoing PCI	Randomization to tailored strategy vs. standard of care	Lower stent thrombosis and ACS rate in tailored strategy
ANTARTIC [121]	2016	VerifyNow	PFT-based dose or drug adjustment in tailored strategy	877 patients undergoing PCI for an ACS	Randomization to tailored strategy vs. standard of care	No differences in 1-year MACE
TROPICAL-ACS [122]	2017	Impedance aggregometry	PFT-based de-escalation strategy after 14 days	2610 patients undergoing PCI for an ACS	Randomization to tailored strategy vs. standard of care	Tailored de-escalation strategy non-inferior to standard of care
CREATIVE [123]	2018	TEG	PFT-based identification of poor responder before randomization	1087 clopidogrel poor responders undergoing PCI	Randomized to higher dose of clopidogrel vs. standard dose of clopidogrel plus cilostazol vs. standard of care	Lower rate of 18-months MACCE with the adjunct of cilostazol

## Data Availability

Data sharing is not applicable to this article.

## Abbreviations

ACS = acute coronary syndrome; ASA = acetylsalicylic acid; CV = cardiovascular; GP IIb/IIIa = glycoprotein IIb/IIIa; LTA = light transmission platelet aggregation; MACCE = major adverse cardiovascular and cerebrovascular event; MACE = major adverse cardiovascular event; MI = myocardial infarction; PCI = percutaneous coronary intervention; PFT = platelet function test; TEG = thromboelastography; VASP = vasodilator stimulated phosphoprotein.
